# Supplementary material for: Artemisia afra and Artemisia annua Extracts Have Bactericidal Activity against Mycobacterium tuberculosis in Physiologically Relevant Carbon Sources and Hypoxia
Source: Pathogens. 2023 Feb 1;12(2):227. doi: 10.3390/pathogens12020227 (PMC9963027; doi:10.3390/pathogens12020227)
Supplement: Supplementary file 1 [file pathogens-12-00227-s001.zip › pathogens-2151589-supplementary.pdf]

SUPPLEMENTAL INFORMATION TO:

***Artemisia afra* and *Artemisia annua* extracts have bactericidal activity against *Mycobacterium tuberculosis* in physiologically relevant carbon sources and hypoxia** by

Bushra Hafeez Kiani, Maria Natalia Alonso, Pamela J. Weathers, Scarlet Shell

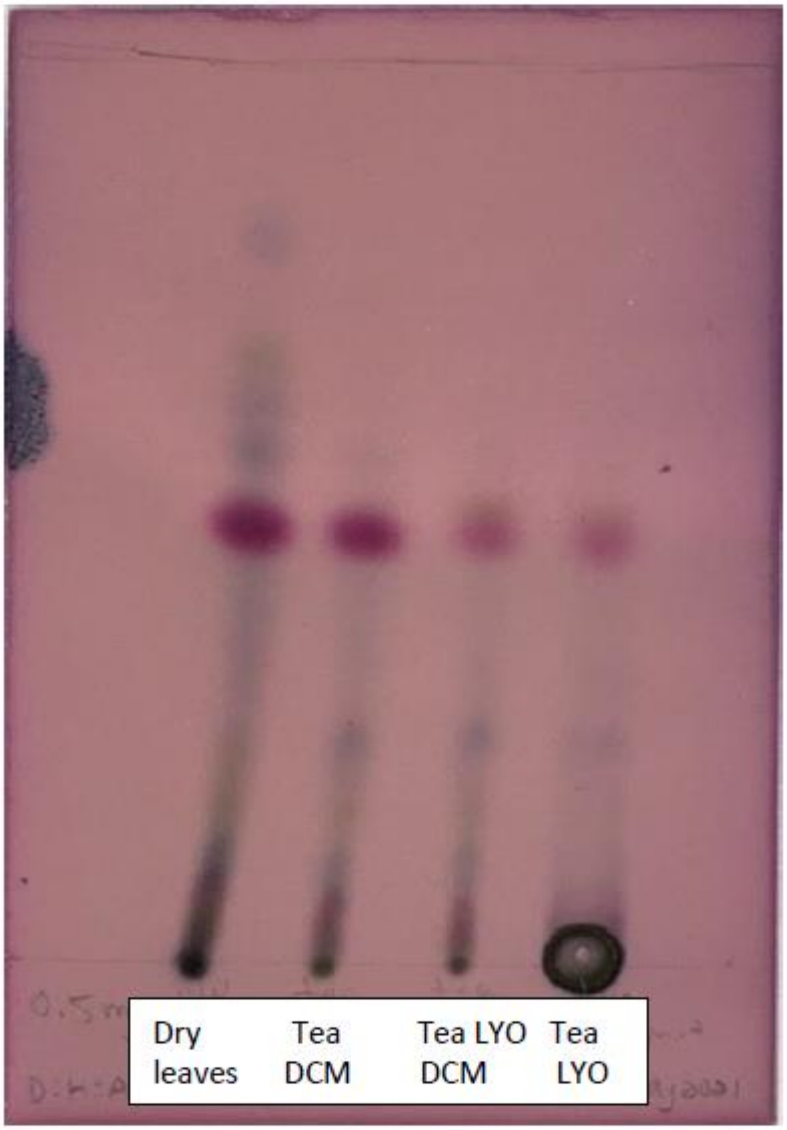

Figure S1. TLC showing loss of components of *Artemisia annua* tea upon lyophilization and reconstitution in water. L to R samples applied were: *A. annua* SAM leaves (2018 crop) DCM extract; SAM leaves at 5 g/L tea (10 min boiling) extracted 1:1 w/DCM; lyophilized tea reconstituted to original volume and then extracted 1:1 w/DCM; lyophilized tea reconstituted but no DCM extracted. All samples were 0.5 mg equivalents for original dried leaf sample. GCMS artemisinin analyses compared to SAM 2018 dried leaves indicated: ~99% recovery from tea, ~60% recovery from reconstituted lyophilized residue.

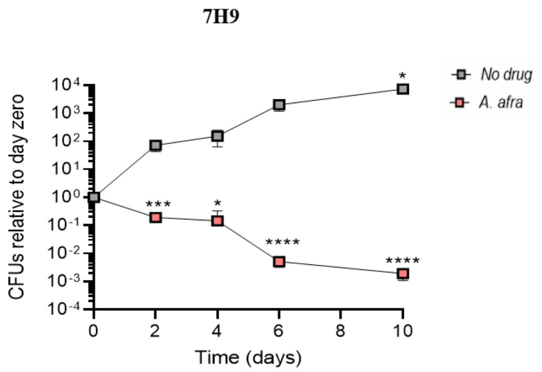
7H9
No drug
A. afra
CFUs relative to day zero
Time (days)

A

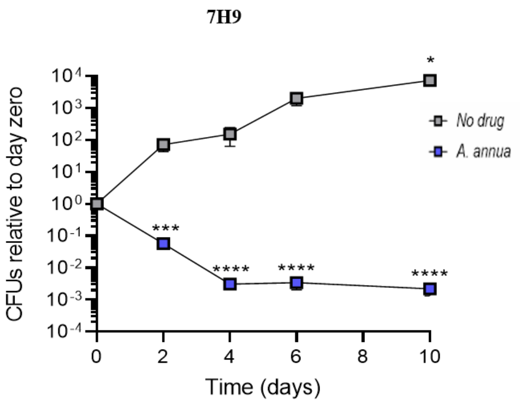
7H9
No drug
A. annua
CFUs relative to day zero
Time (days)

B

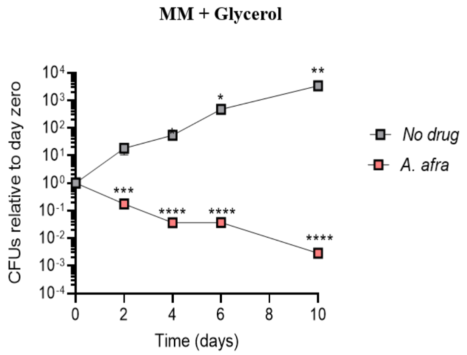
MM + Glycerol
No drug
A. afra
CFUs relative to day zero
Time (days)

C

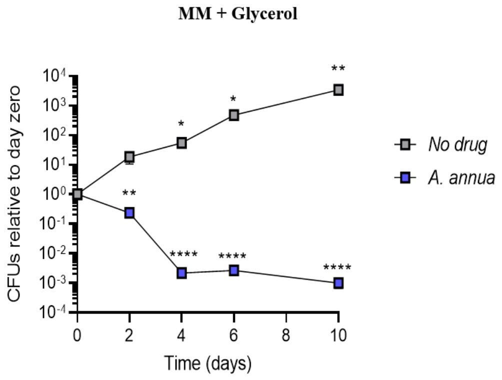
MM + Glycerol
No drug
A. annua
CFUs relative to day zero
Time (days)

D

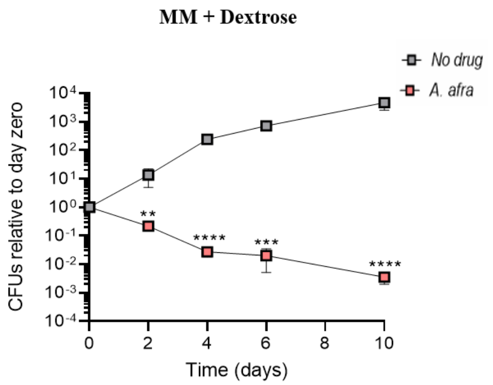
MM + Dextrose
No drug
A. afra
CFUs relative to day zero
Time (days)

E

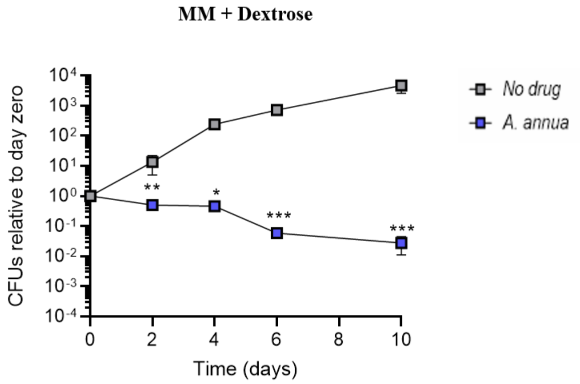
MM + Dextrose
No drug
A. annua
CFUs relative to day zero
Time (days)

F

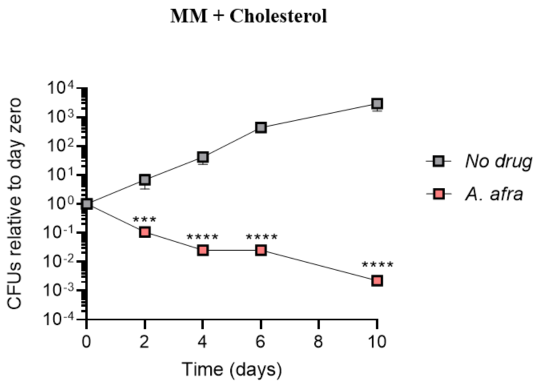

G

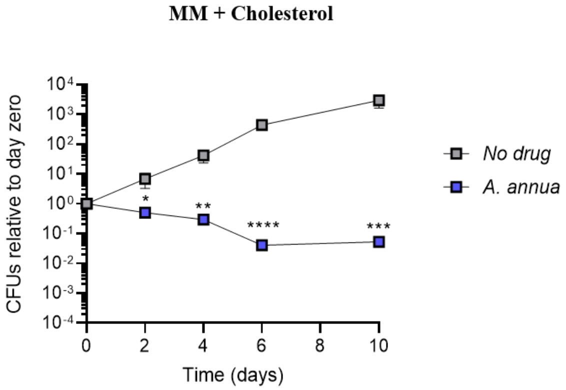

H

Figure S2. *A.afra* and *A.annua* bactericidal activity against *Mtb* in different carbon sources

Figure A-H: Comparative analysis of Mtb growth under different carbon sources (A) *A. afra* in rich medium 7H9 + OADC (0.05% Tween). (B) *A. annua* rich medium 7H9 + OADC (0.05% Tween). (C) *A. afra* in minimal medium containing glycerol as a single carbon source. (D) *A. annua* in a minimal medium containing glycerol as a single carbon source. (E) *A. afra* in minimal medium containing dextrose as a single carbon source. (F) *A. annua* in minimal medium containing dextrose as a single carbon source. (G) MM + Cholesterol *A. afra* (H) *A. annua* in minimal medium containing cholesterol as a single carbon source. N=3; bars = ± SD; * = P<0.05; ** = P<0.01; *** = P<0.001; **** = P<0.0001 for indicated timepoints relative to time 0.
